# Resveratrol Mitigates Cognitive Impairments and Cholinergic Cell Loss in the Medial Septum in a Mouse Model of Gradual Cerebral Hypoperfusion

**DOI:** 10.3390/antiox13080984

**Published:** 2024-08-14

**Authors:** Eric Fagerli, Charles W. Jackson, Iris Escobar, Fernando J. Ferrier, Efrain J. Perez Lao, Isabel Saul, Jorge Gomez, Kunjan R. Dave, Oliver Bracko, Miguel A. Perez-Pinzon

**Affiliations:** 1 Peritz Scheinberg Cerebral Vascular Disease Research Laboratories, University of Miami Leonard M. Miller School of Medicine, Miami, FL 33136, USA; eaf100@students.miami.edu (E.F.); charles.jackson@osumc.edu (C.W.J.); iris.escobar@yale.edu (I.E.); fjf43@students.miami.edu (F.J.F.); ejp134@students.miami.edu (E.J.P.L.); isaul@med.miami.edu (I.S.); jxg5637@med.miami.edu (J.G.); kdave@med.miami.edu (K.R.D.); 2 Department of Neurology, University of Miami Leonard M. Miller School of Medicine, Miami, FL 33136, USA; oliver.bracko@miami.edu; 3 Neuroscience Program, University of Miami Leonard M. Miller School of Medicine, Miami, FL 33136, USA; 4 Department of Biology, University of Miami, Coral Gables, FL 33146, USA

**Keywords:** neuroprotection, basal forebrain, septo-hippocampal pathway, Alzheimer’s disease, hippocampus

## Abstract

Vascular cognitive impairment and dementia (VCID) is the second leading cause of dementia. There is currently no effective treatment for VCID. Resveratrol (RSV) is considered an antioxidant; however, our group has observed pleiotropic effects in stroke paradigms, suggesting more effects may contribute to mechanistic changes beyond antioxidative properties. The main goal of this study was to investigate if administering RSV twice a week could alleviate cognitive declines following the induction of a VCID model. Additionally, our aim was to further describe whether this treatment regimen could decrease cell death in brain areas vulnerable to changes in cerebral blood flow, such as the hippocampus and medial septum. We hypothesized RSV treatments in a mouse model of gradual cerebral hypoperfusion protect against cognitive impairment. We utilized gradual bilateral common carotid artery stenosis (GBCCAS) via the surgical implantation of ameroid constrictor devices. RSV treatment was administered on the day of implantation and twice a week thereafter. Cerebral perfusion was measured by laser speckle contrast imaging, and cognitive functions, including the recognition memory, the spatial working memory, and associative learning, were assessed by novel object recognition (NOR), Y-maze testing, and contextual fear conditioning (CFC), respectively. RSV treatment did not alleviate cerebral perfusion deficits but mitigated cognitive deficits in CFC and NOR after GBCCAS. Despite these deficits, no hippocampal pathology was observed; however, cholinergic cell loss in the medial septum was significantly increased after GBCCAS. This cholinergic cell loss was mitigated by RSV. This study describes a novel mechanism by which chronic RSV treatments protect against a VCID-induced cognitive decline through the preservation of cholinergic cell viability to improve memory performance.

## 1. Introduction

The demographic shift toward an aging population has significantly increased the global prevalence of cognitive impairments, with vascular cognitive impairment and dementia (VCID), primarily stemming from cerebrovascular disease, being identified as a major contributing factor [1,2,3,4]. VCID encompasses a spectrum of cognitive disorders ranging from a mild cognitive impairment to vascular dementia [5]. These conditions arise from ischemic or hemorrhagic vascular events and may occur independently or in conjunction with neurodegenerative processes such as Alzheimer’s disease (AD) [1]. A critical factor contributing to VCID is global cerebral hypoperfusion, often due to carotid artery disease or heart failure [6]. Consequently, animal models that mimic the cerebral hypoperfusion observed in humans are essential for understanding the pathophysiological mechanisms of VCID. To replicate these conditions, various rodent models of chronic cerebral hypoperfusion have been developed, including bilateral common carotid artery (CCA) occlusion in rats, bilateral CCA stenosis in mice or gerbils, and unilateral CCA occlusion in mice [7]. To better replicate the chronic, progressive reduction in global cerebral blood flow (CBF), characteristic of VCID, we employed a model involving the surgical placement of ameroid constrictors (ACs) around the CCAs. These devices gradually narrow the arteries by absorbing water and swelling, thereby providing a predictable and controlled stenosis [8].

Current treatment options for VCID show minimal efficacy and have spurred interest in alternative therapies, including natural compounds like resveratrol [9,10,11]. Resveratrol (trans-3,4,5’-trihydroxy-stilbene, RSV) is a natural polyphenol found in grape skins and seeds and is known for its antioxidant, anti-inflammatory, and anti-apoptotic properties [12]. RSV has significantly increased in its popularity as a dietary supplement. It is a polyphenolic phytoalexin and powerful antioxidant, with its structural composition containing three hydroxyl groups able to scavenge free radicals [13]. Since oxidative stress-induced brain damage is a critical mechanism that has been reported in VCID progression, the antioxidative properties of RSV may offer a strong therapeutic opportunity [14,15]. The resultant aberrations in the cerebrovascular endothelial function developed from VCID may further contribute to cognitive deficiencies. In pathological conditions, endothelial nitric oxide synthase (eNOS) may become uncoupled and consequently transition from generating nitric oxide to producing harmful superoxide radicals [16,17]. RSV may provide a therapeutic avenue to prevent this uncoupling and promote proper eNOS activity, thus counteracting the oxidative stressor production that would lead to cognitive deficits [18].

We and others have demonstrated that RSV preconditioning mimics ischemic preconditioning in the brain, offering protection in animal stroke models (Middle Cerebral Artery Occlusion—MCAO) [19,20,21,22,23]. Furthermore, we have shown that RSV enhances the antioxidant defense mechanisms via Nrf2 [23] and preserves cognitive functions post-cerebral ischemia through the maintenance of synaptic plasticity [20], the enhancement of DNA repair mechanisms [20,24], and the induction of mitochondrial metabolic adaptations [19]. RSV also induces AMPK activation via protein kinase C epsilon, which in turn elevates the mitochondrial levels of Nampt, which is the critical enzyme for catalyzing NAD formation for both the tricarboxylic acid cycle and the electron transport chain [25]. RSV has also been shown to induce neuroprotection by enhancing synaptic plasticity through the activation of Nrf2, a transcription factor that upregulates antioxidant systems and helps to maintain proper mitochondrial coupling and antioxidant protein expression [26,27].

Our recent studies have highlighted the neuroprotective effects of RSV in the basal forebrain (BF) structures, such as the medial septal nucleus and the diagonal band of Broca, following cerebral ischemia [28]. These BF regions are critical, as their cholinergic projections to the cerebral cortex, hippocampal complex, and amygdala play essential roles in cognitive functions [29]. The early and selective loss of cholinergic neurons in these areas is linked to cognitive deficits observed in various neurodegenerative diseases, including AD [30,31]. Despite its significance, the BF has not been extensively studied in VCID models.

As a potential therapeutic, daily RSV administration has been shown to prevent memory declines in murine models of vascular dementia [32,33,34]. Our previous research demonstrated that an intermittent RSV treatment was as effective as daily dosing in reducing infarct volumes following focal cerebral ischemia [21]. This finding suggests that an intensive RSV treatment may not be necessary for cognitive protection in VCID. Therefore, a primary objective of this study was to determine whether a regimen of twice a week RSV treatment can mitigate cognitive declines in a VCID model. Additionally, we aimed to assess whether this regimen can also reduce cell death in brain regions susceptible to alterations in cerebral perfusion, such as the hippocampus and medial septum, as evidenced in our recent focal cerebral ischemia model [28].

## 2. Materials and Methods

### 2.1. Animals

Animal use in this study was approved by the Institutional Animal Care and Use Committee of the University of Miami. Experiments using animals followed the ARRIVE Guidelines and the Guide for the Care and Use of Laboratory Animals of the National Institutes of Health (NIH). Male C57BL/6J mice, aged 8 to 12 weeks (Jackson Laboratories, Farmington, CT, USA), were housed in the facilities of the Department of Veterinary Resources of the University of Miami under a 12 h light–dark cycle with food and water ad libitum. Animals were separated into four groups at random. The two sham groups consisted of n = 11 each, and the two VCID groups consisted of n = 8 each.

### 2.2. Surgical Implantation of Ameroid Constrictor

The ameroid constrictor (Research Instruments SW, Escondido, CA, USA) consists of a stainless-steel casing surrounding a hygroscopic casein material with an internal lumen. The casein component gradually absorbs water and consequently swells, leading to a predictable narrowing of the carotid arterial lumen it encases. The internal diameter, outer diameter, and length of the AC used prior to surgical implantation were 0.75 mm, 3.25 mm, and 1.25 mm, respectively.

Mice were anesthetized with 3% isoflurane and maintained at 1.5% isoflurane in 100% O_2_. Following a midline cervical incision, both CCAs were exposed and freed from their sheaths. A 4-0 silk suture was placed around each CCA, the CCAs were lifted gently by the suture, and ACs were implanted surgically and bilaterally on the CCAs prior to the bifurcation of the external branching. Sham animals received the same surgery without the implantation of ACs. The rectal temperature was maintained between 36.0 °C and 38.0 °C.

### 2.3. Resveratrol Treatment

Animals were treated on the day of surgical implantation within 30 min following surgical implantation and treated twice a week throughout thereafter. A 100% solution of DMSO was used to dissolve trans-resveratrol (Sigma-Aldrich, St. Louis, MO, USA) to give a final concentration of 65 mg/mL and stored in amber tubes at −20 °C. Immediately before use, aliquots were diluted to a 1 mg/mL working solution (1.5% DMSO) with saline and mixed vigorously. A total of 10 mg/kg of RSV or a vehicle (Veh) was injected intraperitoneally in low light to avoid trans/cis isomerization.

### 2.4. Laser Speckle Contrast Imaging

CBF was determined by laser speckle contrast imaging (LSCI) with the RFLSI III Laser speckle Imaging System (RWD, Sugar Land, TX, USA), which enables high-resolution 2-dimensional imaging and has a linear relationship with absolute CBF values [35]. Briefly, animals were anesthetized with 3% isoflurane in 100% O_2_ in an induction chamber. Once anesthetized, 1.5–2% isoflurane in 100% O_2_ was maintained during the imaging of the animal’s exposed skull covered in saline after removing the skin and periosteum, and the animal’s temperature was maintained at around 37 °C via a heating pad. The same size region of interest (2 mm^2^) for the target area measuring CBF was located 1 mm posterior and 2 mm lateral of Bregma. The field of view covered approximately 121 mm^2^ (11 × 11 mm). The following imaging parameters were used: a display rate of 25 Hz, a time constant of 1 s, a camera exposure time of 5 milliseconds, a camera frame rate of 37.59 frames per second, a laser intensity of 100 mA, and a resolution of 2048 × 2048 pixels. One CBF image was generated by averaging numbers obtained from 20 consecutive raw speckle images. After the images were taken, the signal intensities of the CBF in the region of interest were calculated at each time point by the RWD Laser Speckle Imaging System software (Version 01.00.05.19623). Relative cerebral blood flow (rCBF) values are presented as a percentage of the preoperative value. The experiment had a final n = 8–11 for each group.

### 2.5. Histological Evaluation

Histopathology assessments were conducted 33 days following AC implementation. Briefly, animals were anesthetized and perfused with 10 mL of ice-cold saline followed by 10 mL of 4% PFA at a rate of 2 mL/min. Harvested brains were left in 4% PFA overnight before being cryoprotected by immersion in increasing sucrose gradients in 1X PBS (15% and 30%) over the following two days and flash-frozen on liquid nitrogen-cooled isopentane (Sigma-Aldrich) in an optimal cutting temperature medium (VWR). Samples were stored at −80° before sectioning. A Leica CM 1850 cryostat was used to cut 30 μm-thick sections. Free-floating sections were subjected to antigen retrieval with 0.3% SDS for 10 min. Slices were washed for 3 × 5 min with a wash buffer (0.1% Tween-20 in 1X PBS, pH 7.4) and incubated in a blocking solution (10% normal donkey serum, 2% BSA, and 0.8% Triton X-100 in a wash buffer) for 1.5 h at RT. Thereafter, slices were rinsed for 3 × 5 min with a wash buffer and incubated overnight at 4 °C with the primary antibody (goat anti-choline acetyltransferase (ChAT) [1:250], AB144P, Millipore and rabbit anti-NeuN [1:1000], 12943S, Cell Signaling Technology) diluted in a wash buffer containing 2% BSA and 0.1% Triton X-100. Sections were rinsed for 4 × 10 min with a wash buffer and incubated with an Alexa 647-([1:250], A78952, ThermoFisher, Waltham, MA, USA) and an Alexa 568-conjugated secondary antibody ([1:1000], A11057, Invitrogen, MA, USA) and diluted in a wash buffer containing 2% BSA and 5% normal donkey serum for 2 h at RT. Slices were then rinsed for 4 × 10 min with a wash buffer and for 2 × 5 min with 1X PBS, and slices were mounted on Superfrost Plus slides (VWR) with coverslips using Prolong Diamond antifade mounting media (ThermoFisher). Imaging was performed using an inverted Leica Stellaris confocal microscope with a 10x objective (N.A. 0.40). High-magnification images were taken using a 20X-objective (N.A. 0.75) and 3×-zoom setting.

### 2.6. Open-Field Testing

Open-field testing was performed at the habituation stage 24 h before novel object recognition testing. Briefly, mice were handled for 5 of the 7 days (2 min per day) prior to the start of behavioral testing to acclimate the animals to human handling. Mice were then placed in a square open-field arena (43 × 43 × 43 cm) and allowed to explore for 10 min while their behavior was recorded using the EthoVision software (Version 8.5). The total distance moved was used as a measure of general activity and locomotor function, while the animal’s tendency to avoid the center area reflected anxiety related behavioral changes [36,37].

### 2.7. Novel Object Recognition Testing

This test was used to assess a rodent’s affinity for a novel object compared with a familiar object [38,39]. Animals were allowed to freely explore the arena containing two identical objects placed at an equal distance for 10 min. A total of 24 h later, the animals were exposed to the testing arena in the presence of a familiar object and a novel object to test the animal’s memory recognition. Both familiarization and testing occurred for a 10 min period [37,40]. The exploratory behavior (defined as the animal directing its nose at a distance of ≤2 cm from the object) was analyzed via the EthoVision software. The discrimination index was defined as the time spent exploring the novel object divided by the overall exploratory activity on the testing day. The objects and arena were cleaned between each trial using 70% ethanol.

### 2.8. Spontaneous Alternation

To evaluate short-term spatial memory, animals were placed in the start arm of the Y maze and allowed to explore the three-goal arms (A, B, and C) for 8 min [41]. The Y maze consisted of 3 identical arms separated via 120° angles, and each arm had a different color tape and different pattern at the end of each arm. Entrance patterns between arms A, B, and C were scored. Arm entry was scored when the mouse placed its 4 paws inside the arm. Spontaneous alternation was determined upon entry into three arms from consecutive selections of three sets (e.g., A–B–C, B–C–A, and C–A–B). The alternation behavior was calculated using the following equation: % alternation = ([Number of alternations]/[Number of total arm entries − 2]) × 100.

### 2.9. Contextual Fear Conditioning

To evaluate contextual fear memory, mice were tested using a contextual fear conditioning paradigm. Briefly, mice were placed in a contextual fear conditioning box for 340 s before 0.6 mA of a current was administered for 2 s to cause a foot shock. The animals remained in the box for 30 additional seconds following the foot shock. Twenty-four hours later, the animals were placed in the box for 480 s with no shocks. The percentage of time spent freezing prior to the shock on day 1 was subtracted from the percentage of time spent freezing on day 2 to determine contextual fear learning and memory. The freezing response was analyzed by the computer software FreezeFrame (Version 3.15).

### 2.10. Statistical Analysis

Mice were randomly assigned to groups. Blinding was incorporated into the experimental design and methodology for all experiments; double blinding was performed so that the researchers were blind during the administration of treatment, the conduction of experiments, and the analysis of data. Tests for data normality and a statistical analysis were conducted with the use of the Prism 9.2.0 software (GraphPad, Boston, MA, USA). For all representations, data are presented with the mean and error bars ± standard errors. Statistical tests included two-way ANOVA for behavioral testing and immunostaining cell counts and repeated measure two-way ANOVA for the analysis of cerebral perfusion. The Bonferroni correction was utilized for post hoc testing. *p*-values of *p* < 0.05 were considered significant. Sample sizes were determined from pilot studies or previous experiments conducted in the lab. When needed, a power analysis determined the effect sizes using the G*power 3.1 software [42]. An α value of 0.05 and a β value of 0.8, with an effect size of 1.1, were utilized based on previous experiments.

## 3. Results

### 3.1. Resveratrol Does Not Ameliorate Reductions in Cerebral Blood Flow in the VCID Model

**Figure 1:**
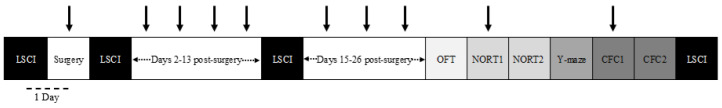
Experimental schematic for in vivo RSV treatments, LSCI measurements, and behavioral tests. LSCI was performed the day prior to AC or sham surgery and then 1, 14, and 33 days following surgery. RSV was administered on the day that the surgery occurred as well as twice per week thereafter, as indicated by the arrows. Behavioral testing occurred on days 27 through 32 following the surgery and consisted of open-field testing (OFT), two days of novel object recognition testing (NORT), Y-maze testing, and two days of contextual fear conditioning (CFC) testing. Arrows indicate days of RSV administration.

Due to previous reports indicating that RSV improves cerebral perfusion via angiogenesis [43,44], we first tested whether RSV was able to mitigate rCBF reductions following AC placement. We hypothesized that RSV treatment following the induction of cerebral hypoperfusion would mitigate global rCBF deficits through RSV-mediated alterations in vessel formation. To evaluate whether RSV can mitigate rCBF reductions, we compared the global rCBF in four groups: Sham + Veh, VCID + Veh, Sham + RSV, and VCID + RSV, as described in Figure 1.

**Figure 2:**
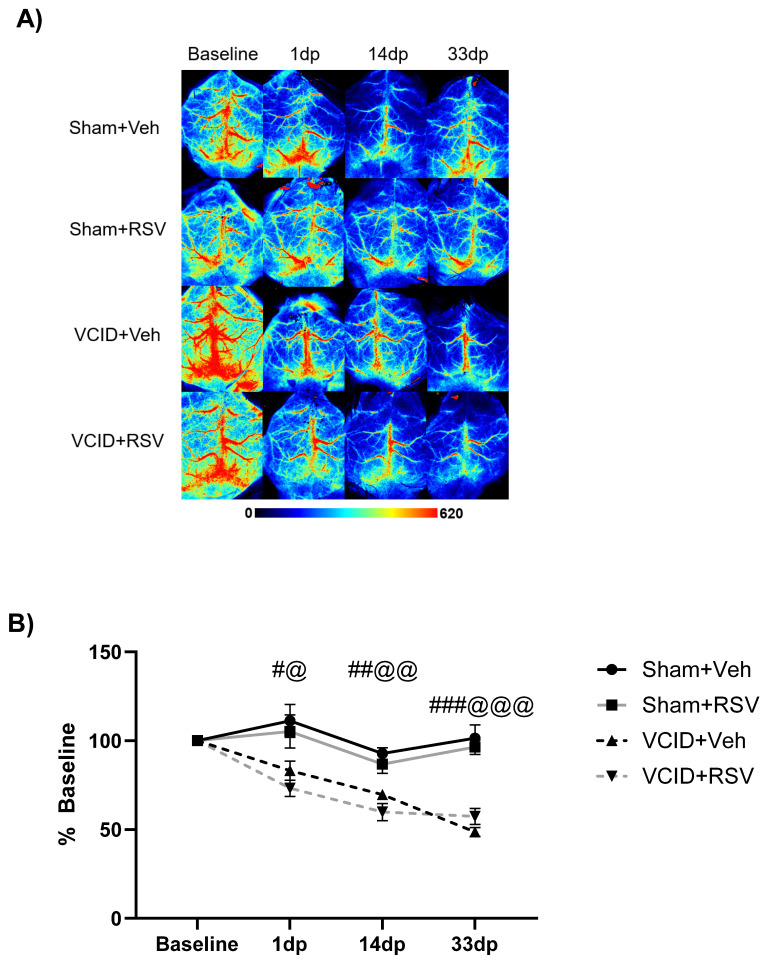
Resveratrol does not ameliorate deficits in cerebral blood flow in the VCID model. (**A**) Representative LSCI images in the four treatment groups. (**B**) Relative cerebral perfusion rates for all groups relative to baseline. Data are expressed as mean ± SEM. n = 8 for each of the VCID groups and 11 for each of the sham groups. dp = days post-surgery. # indicates significance between the VCID + Veh group compared to the Sham + Veh group, while @ indicates significance between the VCID + RSV and Sham + RSV groups. # and @ *p* < 0.05. ## and @@ *p* < 0.01. ### and @@@ *p* < 0.001.

The average rCBF values for each imaging session following the surgery are shown in Table 1. There were significant differences in the rCBF 1 day post-surgery (83 ± 5% vs. 111 ± 9%, *p* < 0.05); 14 days post-surgery (70 ± 2% vs. 93 ± 3%, *p* < 0.01); and 33 days post-surgery (49 ± 3% vs. 101 ± 5%, *p* < 0.001) in the VCID + Veh group compared to the Sham + Veh group (Figure 2A,B). Similarly, in the RSV-treated groups, there was a significant difference in the rCBF 1 day post-surgery (73 ± 4% vs. 105 ± 9%, *p* < 0.05); 14 days post-surgery (59 ± 4% vs. 86 ± 5%, *p* < 0.01); and 33 days post-surgery (57 ± 4% vs. 96 ± 4%, *p* < 0.001) in the VCID + RSV group compared to the Sham + RSV group (Figure 2B).

However, no significant differences in the rCBF were observed between the vehicle and RSV treatment within the VCID animals at 1 day post-surgery (73 ± 5% vs. 83 ± 5%, nonsignificant (ns)); 14 days post-surgery (60 ± 5% vs. 70 ± 2%, ns); and 33 days post-surgery (57 ± 5% vs. 49 ± 3%, ns) (Figure 2B). This indicates that RSV does not ameliorate deficits in the global rCBF in the VCID model.

### 3.2. General Activity/Locomotor Function, Anxiety-Related Behavior, and Spatial Working Memory Are Not Affected by VCID

**Figure 3:**
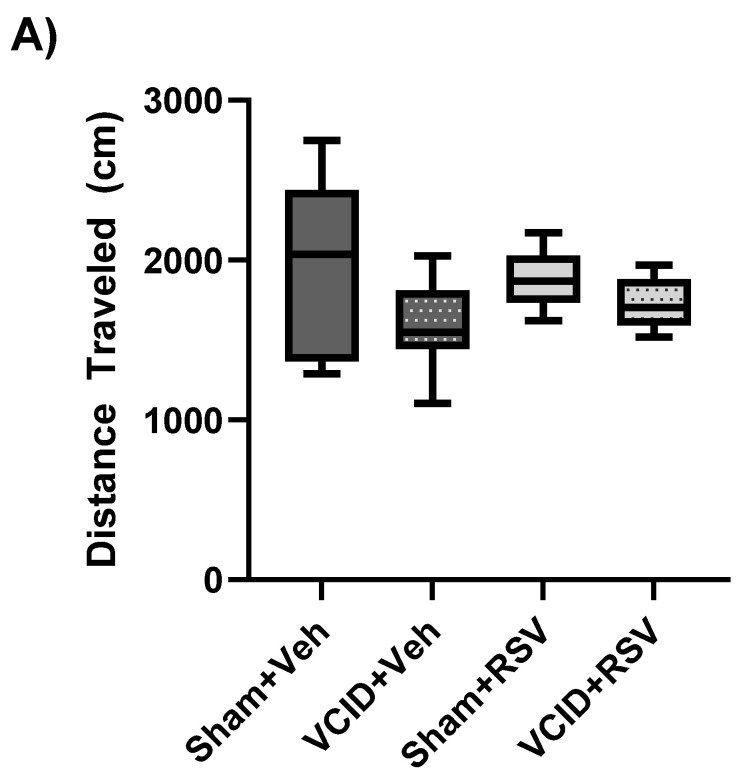

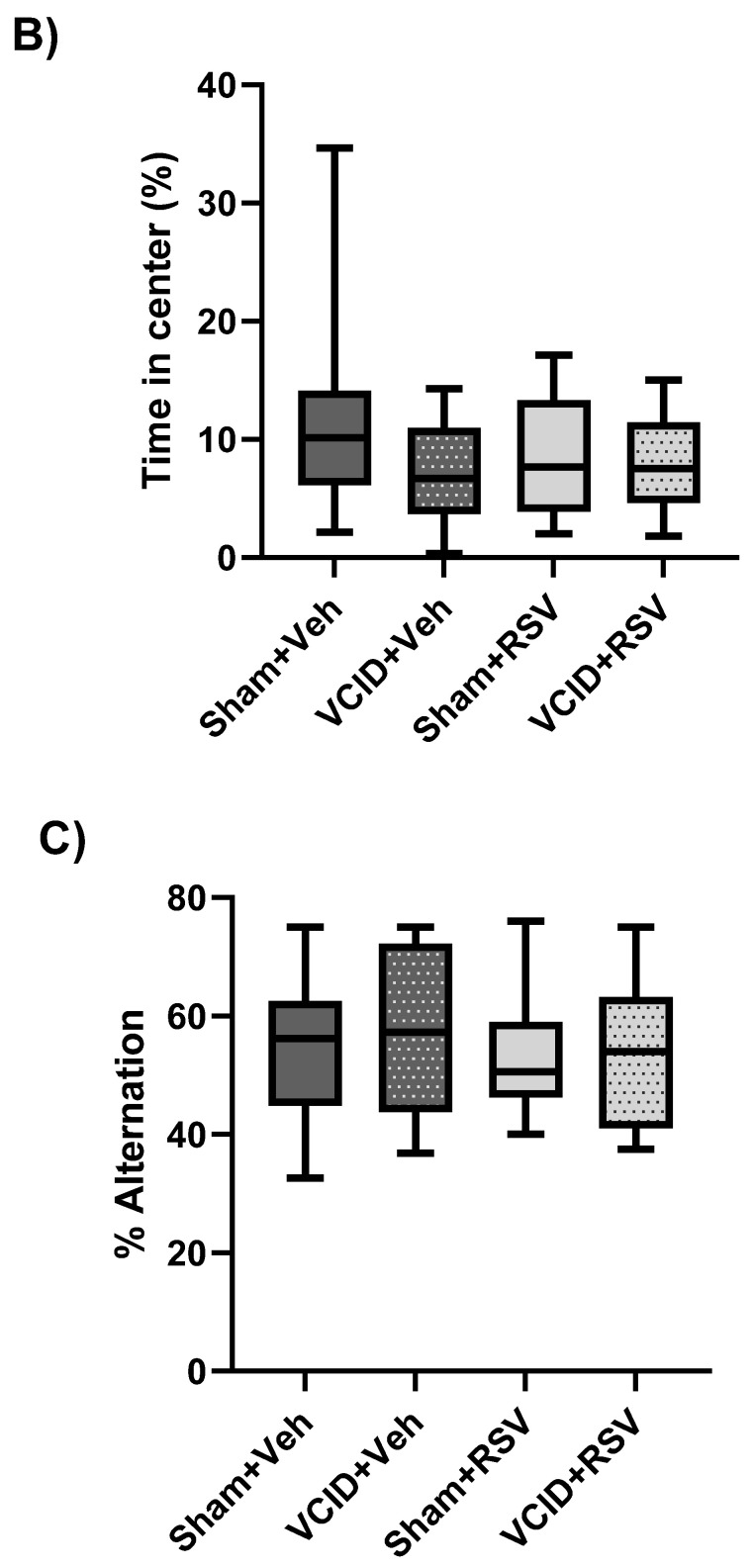
General activity/locomotor function, anxiety-related behavior, and spontaneous alternation via Y-maze testing are not affected by VCID. An analysis was performed on (**A**) the distance traveled, (**B**) the time spent in the center of the open field, which was found among the groups, and (**C**) spontaneous alternations in Y-maze testing, which were found. Data are expressed as a mean ± SEM. n = 8 for each of the VCID groups and 11 for each of the sham groups.

The open-field test was utilized to evaluate the general activity and locomotor function as well as anxiety-related behavior. Additionally, it served as a screening procedure to ensure there were no outlier behavior traits in the experimental animals in further cognitive testing. No significant differences were found among the Sham + Veh, Sham + RSV, VCID + Veh, or VCID + RSV groups (2069 ± 210, 1899 ± 92, 1513 ± 192, and 1713 ± 96, in cm, respectively) regarding the general activity/locomotor function, as determined by an analysis of the total distance traveled (cm) during testing (Figure 3A). No significant differences were found among the Sham + Veh, Sham + RSV, VCID + Veh, or VCID + RSV groups (12 ± 4, 9 ± 1, 7 ± 2, and 10 ± 2, % of time spent in center, respectively) regarding anxiety-related behavior, as determined through the percentage of time spent in the center of the open field (Figure 3B). These findings suggest that VCID surgery as well as the RSV treatment does not affect the general activity, locomotor function, or anxiety-related behavior in the VCID model. Furthermore, these results suggest that any differences in behavioral observations cannot be attributed simply to gross motor differences brought about from VCID.

The hippocampus and entorhinal cortex play an important role in episodic memory and spatial navigation [45]. While reports of spatial working memory deficits have varied in animal models of VCID related to the paradigm timeframe [46,47,48], we hypothesized that our model of gradual carotid artery stenosis would induce spatial working memory deficits that would be ameliorated via RSV treatment. To assess discrepancies in spatial working memory, animals underwent Y-maze testing for spontaneous alternations. No significant differences in spontaneous alternation behavior were observed among the Sham + Veh, Sham + RSV, VCID + Veh, or VCID + RSV groups (58 ± 3, 53 ± 3, 66 ± 5, and 53 ± 5, % alternation, respectively) (Figure 3C). From these results, we determined that our model of VCID via a gradual AC stenosis of the CCAs did not induce spatial working memory deficits over the time course of our paradigm.

### 3.3. Resveratrol Mitigates Impairments in Long-Term Object Recognition Memory and in Contextual Fear Memory That Arise from VCID

**Figure 4:**
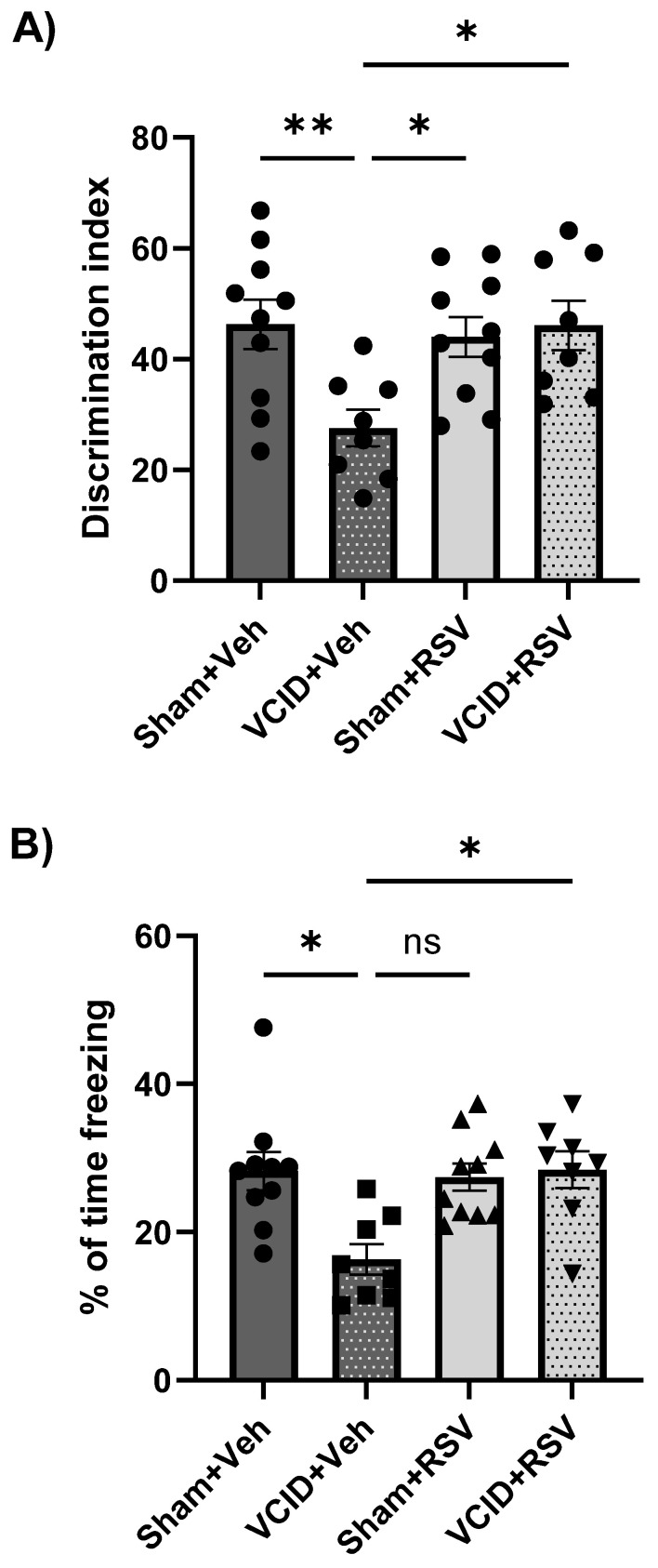
Impairments in object recognition memory and contextual fear memory caused by VCID were reduced by resveratrol treatments. (**A**) VCID-induced impairment in recognition memory, which was ameliorated via resveratrol treatments. (**B**) VCID-induced impairment in contextual fear memory, which was prevented by resveratrol treatments. Data are expressed as a mean ± SEM. n = 8 for each of the VCID groups and 11 for each of the sham groups. * *p* < 0.05. ** *p* < 0.01.

Novel object recognition testing was used to evaluate the hippocampal function and long-term recognition memory, as an animal’s preference to explore a novel object displays the use of the learning and recognition memory regarding familiar object exposure. We hypothesized that the recognition memory would be disrupted in VCID animals due to a decreased perfusion but that RSV treatment would ameliorate these impairments. VCID caused a significant loss of object recognition, and this loss of object discrimination was prevented in animals treated with RSV (Figure 4A). The Sham + Veh, Sham + RSV, and VCID + RSV groups had significant increases in their discrimination index compared to the VCID + Veh group (52 ± 5, 53 ± 3, and 56 ± 4 vs. 29 ± 6, expressed as the % of time exploring the novel object vs. the % of time exploring the novel object and the familiar object, *p* < 0.05 and *p* < 0.01, respectively) (Figure 4A).

It is widely recognized that the hippocampal function is crucial for acquiring and expressing contextual conditioned fear memories [49]. Previous research has shown that resveratrol can protect against ischemia-related hippocampal synaptic dysfunctions [3] and contextual fear memories [28]. Therefore, we hypothesized that RSV treatment in our VCID model would ameliorate contextual fear memory deficits. Significant differences were found in freezing responses between the Sham + Veh, Sham + RSV, and VCID + RSV vs. VCID + Veh groups (28 ± 2, 27 ± 2, and 28 ± 3 vs. 16 ± 2, with the freezing index expressed as %, *p* < 0.05, respectively) (Figure 4B).

### 3.4. Hippocampal CA1 Pyramidal Cells Are Unaffected in the VCID Model

**Figure 5:**
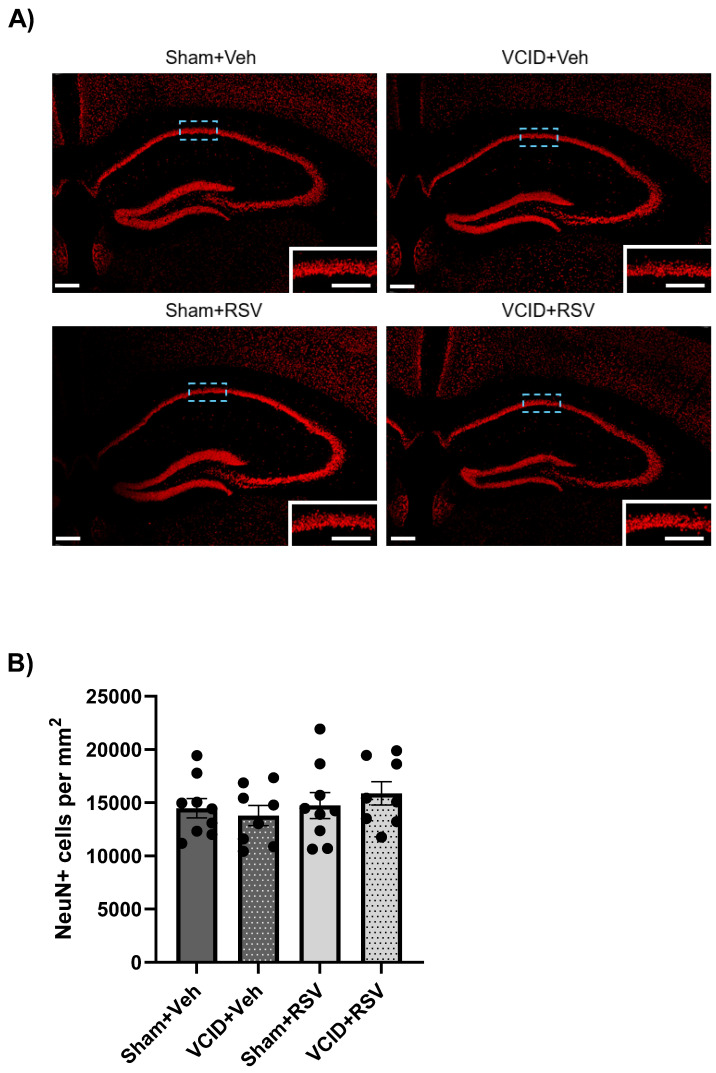
The hippocampal neuronal count in the CA1 pyramidal layer was unaffected by the VCID model, as shown in (**A**) the representative images and the (**B**) the quantification of the CA1 pyramidal layer. Scale bar = 200 μm (main image) or 100 μm (inset image). Data are expressed as a mean ± SEM. n = 8 for each of the VCID groups and 9 for each of the sham groups.

Previous reports of the gradual CCA stenosis model from AC implantation have indicated no significant reductions in the neuronal population of CA1 [11]. However, due to the hippocampal CA1 region’s importance in spatial and contextual memory [50], we assessed the VCID model’s influence on the neuronal survival of hippocampal CA1. No significant differences were found in the CA1 neuronal counts in the VCID animals compared to the Sham animals, regardless of the RSV or Veh treatments (the Sham + Veh, Sham + RSV, VCID + Veh, or VCID + RSV groups: 14,476 ± 908, 13,791 ± 956, 14,732 ± 12,234, and 15,868 ± 1093, CA1 neurons per mm^2^, respectively) (Figure 5B).

### 3.5. Resveratrol Mitigates VCID-Induced Medial Septal Cholinergic Neuronal Loss

**Figure 6:**
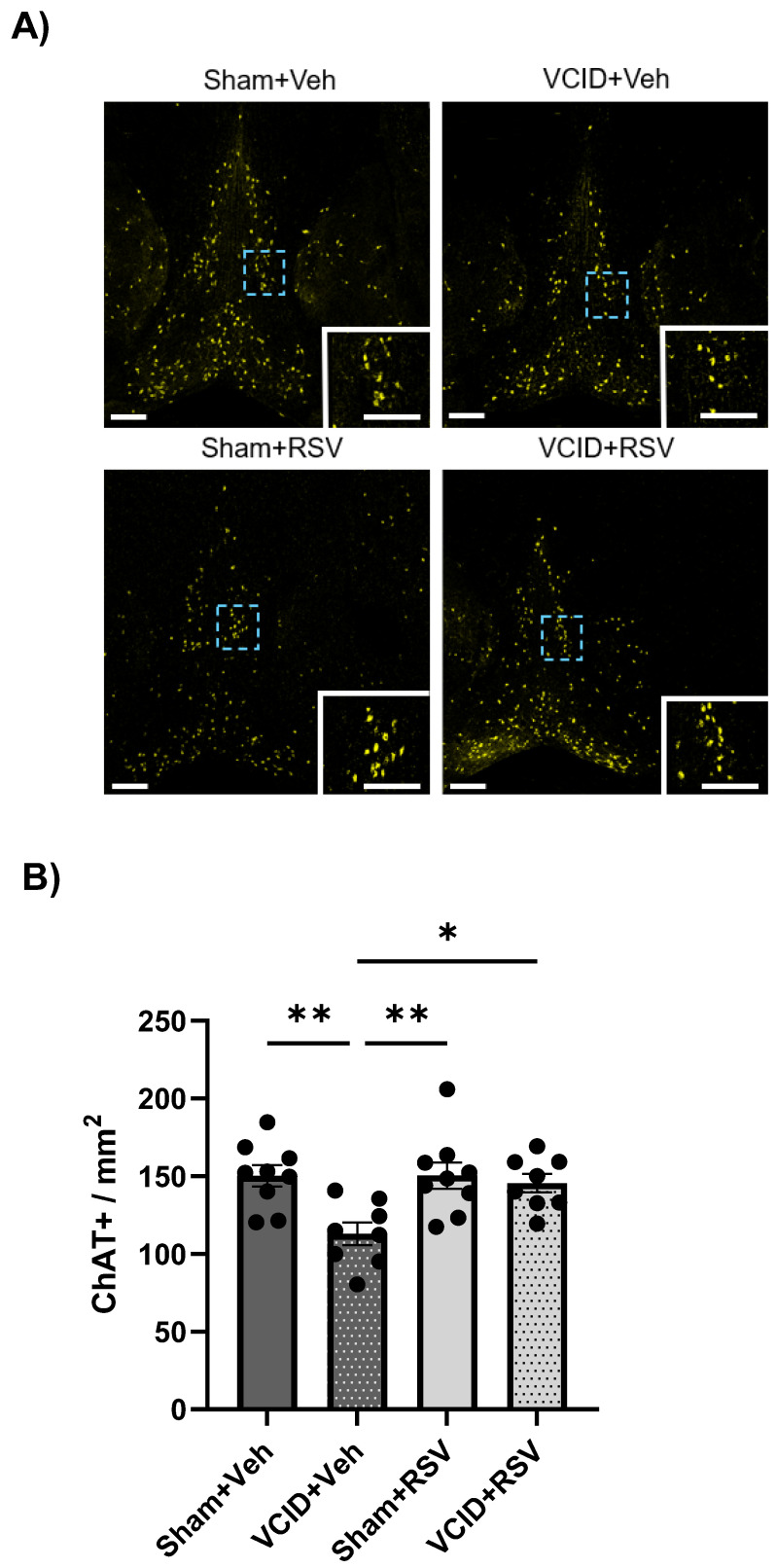
Resveratrol mitigates medial septal cholinergic neuronal loss following 33 days of VCID. (**A**) Maximum intensity projection images showing expression of ChAT-positive neurons (yellow) in the medial septum at Bregma +0.88 mm. (**B**) Quantified number of ChAT-positive cells in the full medial septum. Scale bar = 200 μm (main image) or 100 μm (inset image). Data are expressed as a mean ± SEM. n = 8 for each of the VCID groups and 9 for each of the sham groups. * *p* < 0.05. ** *p* < 0.01.

In a previous study, we found that RSV mitigated cognitive deficits following focal cerebral ischemia and that this protection was correlated with the preservation of cholinergic neurons in the septal nuclei [28]. To assess whether VCID induces pathology in cholinergic neurons in the septal nuclei and whether RSV could mitigate this pathology, we analyzed the cholinergic populations in the medial septum at +0.88 mm relative to the Bregma (Figure 6A). Significant differences were found in the medial septal cholinergic populations at +0.88 relative to the Bregma among the Sham + Veh, Sham + RSV, and VCID + RSV groups vs. the VCID + Veh group (150 ± 7, 151 ± 9, and 146 ± 6 vs. 113 ± 7, ChAT + neurons/mm^2^, *p* < 0.05, respectively) (Figure 6B). These results support our hypothesis that RSV ameliorates cognitive impairments in VCID via mitigating cholinergic neuronal losses in the medial septum.

## 4. Discussion

In the current study, we utilized an animal model of hypoperfusion-induced VCID via the implantation of hygroscopic AC on the CCAs, inducing gradual stenosis that replicates gradual CCA stenosis, leading to global cerebral hypoperfusion in clinical cases. Additionally, we demonstrated that a regimen of resveratrol treatment (10 mg/kg twice a week) following the induction of global hypoperfusion in mice protects against cognitive declines after 27 days. This protection against cognitive declines correlated with the amelioration of cholinergic cell death in the medial septum. These results suggest that the projections from the septo-hippocampal pathway were linked to cognitive and oscillatory hippocampal activities [45]. To the best of our knowledge, this is the first report depicting RSV’s ability to provide protection to cholinergic populations in the medial septum in a chronic, gradual hypoperfusion model.

As described above, a variety of rodent models of chronic cerebral hypoperfusion have been established; however, due to differences in the cerebrovasculature of rodents and humans, certain limitations have been observed regarding the mimicry of clinical hypoperfusive VCID scenarios in murine models [11,51,52]. A model utilizing bilateral CCA occlusion in rats generated white matter lesions in the optic tract that prevented optically based behavioral testing [51]. Mouse models of a fixed CCA stenosis result in a sharp decline in the CBF following surgeries, with a gradual recovery of the CBF over time, which fails to replicate typical chronic hypoperfusive VCID in clinical settings [11,51]. Thus, in the current study, we used a model of VCID using gradually constricting CCAs to induce reproducible, gradual reductions in the rCBF [11].

However, while we expected RSV to mitigate rCBF declines in our VCID model due to its angiogenic properties [43,44], we found that no significant recovery of the rCBF occurred in the RSV-treated animals (Figure 2). RSV’s angiogenetic effects may be reduced in VCID due to decreases in global cerebral perfusion, thus reducing the distribution of RSV throughout the brain. Additionally, while angiogenesis has been seen with RSV treatment following cerebral ischemia, the energetic demands for the angiogenic processes remain unknown [53].

Anxiety and motor function disparities are sometimes symptoms of patients with VCID, but the relationship between VCID, anxiety, and motor function remains unclear [54,55,56]. In this study, we assessed RSV’s impact on anxiety-related behavior and general locomotion utilizing open-field testing and determined that our model of VCID did not induce anxiety or locomotive deficits (Figure 3A,B). The manifestation of anxiety and motor deficits is likely related to the severity, size, and location of lesion developments from VCID. The severity of afflictions likely results from differences in the degree of cerebrovasculature damage and normal nonuniformity in cerebrovasculature between individuals.

Our group has provided significant data and examples of studies supporting the viability of RSV as a protective agent against neurological disorders VCID [57]. To assess cognitive declines in our VCID model and whether RSV could mitigate these deficits, we utilized an array of cognitive tests, such as novel object recognition testing, Y-maze spontaneous alternation testing, and contextual fear memory testing.

Other models of non-gradual bilateral carotid artery stenosis have shown deficits in object recognition memory [58], and our group has previously shown that RSV preconditioning ameliorates cognitive declines in a model of MCAO [28]. We found that RSV treatment significantly mitigated long-term recognition memory deficits in animals with VCID (Figure 4A).

Additionally, we performed Y-maze spontaneous alternation testing and observed no disturbances in the short-term working memory (Figure 4). The lack of deficits in the hippocampal CA1 cell loss (Figure 5) provides an explanation for the lack of spatial deficits observed in the VCID model. While our model accurately mimicked gradual, global hypoperfusion via a stenosis of the CCAs, there was an inherent limitation of the total global rCBF reduction, in that the cerebral posterior influx from the vertebral arteries was not reduced [59]. The lack of significant neuronal cell loss was similarly observed by Hattori et al. [11]. It is possible that CA1 pathology takes longer to emerge than the allotted time in our paradigm.

We also assessed RSV’s effect on the long-term contextual fear memory using contextual fear conditioning. We observed that deficits in the contextual fear memory were mitigated by RSV treatment (Figure 4B), which corroborates our groups previous observation that RSV ameliorates the contextual fear memory following MCAO in rats [28]. Interestingly, our model contrasts a model of unilateral CCA occlusion to mimic VCID that did not induce contextual fear memory deficits [60]. The dorsal hippocampus of rodents is largely important in proper acquisition and recall regarding contextual fear [49], and it is also the CA1 of this region of the hippocampus where cholinergic inputs from the medial septum project [45]. However, we did not observe CA1 pathology in our study. A possible explanation for VCID-induced cognitive deficits comes from selective lesion studies that demonstrated that cholinergic projections originating from the MSN/DB are critical for the fear memory [61,62]. This study suggests that the cholinergic pathology we observed in the current study in the MSN/DB may be at the root of CFC deficits, while RSV-induced cholinergic MSN/DB protection may explain CFC improvements (Figure 6).

As the degeneration and/or loss of cholinergic neurons in the BF is linked to cognitive impairment and is a hallmark of Alzheimer’s disease and vascular dementia [31,63], our findings suggest that impaired cholinergic signaling in the medial septum may play a causal role in the development of cognitive deficits. These findings indicate that RSV treatment could be an effective therapeutic approach for combating VCID-induced cognitive declines, likely by maintaining the cholinergic function between key brain structures involved in memory processing. A model of VCID utilizing an AC on one CCA and the ligation of the other via sutures was also able to show a cholinergic cell loss in the medial septum; however, significant variations were found in their ligated versus gradually constricted hemispheres due to a variation in the surgical procedure between the CCAs [64].

To the best of our knowledge, ours is the first study indicating cholinergic cell loss in a global hypoperfusion model of VCID.

To date, numerous rodent models of VCID have been established for chronic cerebral hypoperfusion models, including bilateral CCA occlusion in rats, bilateral CCA stenosis in gerbils, unilateral CCA occlusion in mice, and bilateral CCA stenosis in mice [10]. However, inherent limitations with models of VCID have been observed regarding the mimicry of clinical hypoperfusive VCID scenarios in murine models [11,51,52]. Additionally, RSV has, to this point, encountered stalling regarding any aspect of the treatment of dementia in clinical trials, even though preclinical work shows strong promise. Derivatives of the chemical formulation of RSV may offer a different therapeutic avenue for future pursuit.

Taken together, this study demonstrates that RSV ameliorates cognitive deficits resulting from VCID in a non-perfusion-dependent manner through the neuroprotection of cholinergic populations in the medial septum. Exactly how RSV treatments promote the protection of these cholinergic neurons warrants further investigation. While the mechanisms behind the RSV-mediated survival of medial septal cholinergic cells are not yet understood, based on our prior findings on the RSV effects on metabolic adaptations that enhance resistance to ischemic insults, we surmise that this is at the core of the RSV mechanism. Mechanistic neuroprotection provided by RSV likely involves a plethora of mechanisms as opposed to a unilateral pathway of action. For example, our lab has previously shown that RSV preconditioning increases acetyl-CoA availability in mitochondria [2]. Our group’s previous findings indicating that RSV activates AMPK through protein kinase C epsilon to increase integral coenzymes for energy production in the mitochondria, as well as our findings that RSV activates Nrf2 to maintain mitochondrial coupling and antioxidant systems, may contribute to the observed neuroprotection in this study [17,19]. RSV’s ability to modulate mitochondrial metabolism could potentially offer a way to counteract reductions in metabolite supply during VCID-related pathological insults; however, further studies are necessary to investigate this possibility.

One potential mechanism of RSV is by targeting one of its best-known targets, SIRT1, a sirtuin involved in lifespan extension across various organisms, including mammals [65,66,67]. SIRT1 achieves this by deacetylating histones and non-histone targets such as TAFI68, MEF2, NF-κB, the tumor suppressors p53 and p73, the DNA repair factor Ku70, and FOXO transcription factors [65,68]. The importance of sirtuin levels is underscored by their association with the progression of Alzheimer’s disease (AD) [69]. Specifically, changes in the expression levels of SIRT1, SIRT3, and SIRT5, as well as their subcellular redistribution in neurons, have been observed in different brain regions of AD patients. In particular, SIRT1 levels are reduced in both aging and AD [70]. Moreover, a decrease in the SIRT1 expression correlates with the local accumulation of tau and amyloid-beta deposits, which are hallmarks of AD [69].

These findings suggest that declines in SIRT1 may contribute to multiple pathological pathways leading to AD and vascular cognitive impairment and dementia (VCID). While the role of SIRT1 has been extensively studied in AD, its involvement in VCID has not been thoroughly investigated. Thus, RSV’s protective effects against cognitive impairment may be linked to its influence on sirtuin pathways, particularly SIRT1, offering potential therapeutic insights for neurodegenerative diseases beyond AD, including VCID. Future studies in our laboratory will address these potential mechanisms.

RSV protected the cholinergic neurons in the septal nuclei in the GBCCAS model. Interestingly, cholinergic dysfunction in the basal forebrain has been ameliorated through the inhibition of oxidative stress [71]. Furthermore, it is well-established that cholinergic and GABAergic contributions to the septo-hippocampal interaction are crucial for supporting theta rhythmogenesis and regulatory activities, which facilitate hippocampal long-term potentiation processes [45,72]. RSV has been shown to provide neuroprotection by enhancing synaptic plasticity through the activation of Nrf2 [26,27], a key component of the cell’s antioxidant defense mechanisms, which is essential for RSV’s neuroprotective effects [23].

Cholinergic-specific lesions in the medial septum have been demonstrated to reduce theta activity in the hippocampus [72]. Therefore, we hypothesize that RSV’s ability to mitigate cholinergic cell loss in the medial septum may help prevent disturbances in theta rhythm activities in the hippocampus. While some clinical studies have explored the effects of RSV treatments, further functional understanding and validation of its benefits are necessary. Future studies should illuminate these potential mechanisms and provide a clearer understanding of RSV’s therapeutic potential.

## Figures and Tables

**Table 1:** Average rCBF values for each imaging session following sham or VCID surgery. Data are expressed as mean ± SEM. Graphical depiction of relative cerebral perfusion and of significance is presented in Figure 2B.

	Sham + Veh	VCID + RSV	Sham + RSV	VCID + Veh
**Day 1**	111 ± 9%	73 ± 4%	105 ± 9%	83 ± 5%
**Day 14**	93 ± 3%	59 ± 4%	86 ± 5%	70 ± 2%
**Day 33**	101 ± 5%	57 ± 4%	96 ± 4%	49 ± 3%

## Data Availability

Data are contained within the article.

## Abbreviations

VCID	Vascular cognitive impairment and dementia
RSV	Resveratrol
GBCCAS	Gradual bilateral common carotid artery stenosis
NOR	Novel object recognition
CFC	Contextual fear conditioning
AD	Alzheimer’s disease
CCA	Common carotid artery
CBF	Cerebral blood flow
CFC	Contextual fear conditioning
AC	Ameroid constrictor
eNOS	Endothelial nitric oxide synthase
MCAO	Middle cerebral artery occlusion
BF	Basal forebrain
ChAT	Choline acetyltransferase
OFT	Open field testing
NORT	Novel object recognition testing
